# Lessons learned from merging wet lab experiments with molecular simulation to improve mAb humanization

**DOI:** 10.1093/protein/gzy009

**Published:** 2018-05-11

**Authors:** L Schwaigerlehner, M Pechlaner, P Mayrhofer, C Oostenbrink, R Kunert

**Affiliations:** 1 Department of Biotechnology, University of Natural Resources and Life Sciences, Muthgasse 18, Vienna, Austria; 2 Department of Material Sciences and Process Engineering, University of Natural Resources and Life Sciences, Muthgasse 18, Vienna, Austria

**Keywords:** antibody humanization, binding affinity, conformational clustering, GROMOS, molecular dynamics

## Abstract

Humanized monoclonal antibodies (mAbs) are among the most promising modern therapeutics, but defined engineering strategies are still not available. Antibody humanization often leads to a loss of affinity, as it is the case for our model antibody Ab2/3H6 (PDB entry 3BQU). Identifying appropriate back-to-mouse mutations is needed to restore binding affinity, but highly challenging. In order to get more insight, we have applied molecular dynamics simulations and correlated them to antibody binding and expression in wet lab experiments. In this study, we discuss six mAb variants and investigate a tyrosine conglomeration, an isopolar substitution and the improvement of antibody binding towards wildtype affinity. In the 3D structure of the mouse wildtype, residue R94h is surrounded by three tyrosines which form a so-called ‘tyrosine cage’. We demonstrate that the tyrosine cage has a supporting function for the CDRh3 loop conformation. The isopolar substitution is not able to mimic the function appropriately. Finally, we show that additional light chain mutations can restore binding to wildtype-comparable level, and also improve the expression of the mAb significantly. We conclude that the variable light chain of Ab2/3H6 is of underestimated importance for the interaction with its antigen mAb 2F5.

## Introduction

In 1984, Niels K. Jerne received the Nobel Prize in Physiology or Medicine for his hypothesis of the immune network (Uhr, 1984). He proposed that antibodies are able to recognize and interact with each other and not uniquely with foreign antigens (Jerne, 1974). Such interactions can elicit the generation of anti-idiotypic antibodies (Ab2) directed against the paratope of a first antibody (Ab1). Thereby, Ab2s are able to form an antigen mimicry of the epitope of Ab1 and could potentially be used as vaccines (Jerne *et al.*, 1982; Fields *et al.*, 1995). Ab2s are most often from murine origin generated by immunization with Ab1 Fab fragments with all potential risks of harmful side-effects in human application (Schroff *et al.*, 1985; Shawler *et al.*, 1985). The murine monoclonal antibody Ab2/3H6 was generated to block the binding of human anti HIV-1 Ab1 2F5 (Kunert *et al.*, 2002) such that it may mimic the HIV-1 antigen. It was further used as model Ab2 for humanization approaches (Gach *et al.*, 2007; Mader and Kunert, 2010). In a rational approach referred to as superhumanization, mouse complementarity determining regions (CDRs) were grafted onto human germline frameworks preserving the canonical structure class (Tan *et al.*, 2002; Hwang *et al.*, 2005). The superhumanization led to a complete loss of binding affinity which was partially restored by a single human-to-mouse backmutation (T94hR; refers to T98hR in Margreitter *et al.*, 2016, mutant BM07). This residue was selected by the synergistic combination of sequence analyses of antibody framework regions and structural information using molecular dynamics simulations. A conglomeration of tyrosine residues surrounding the residue 94h was identified, which was termed the ‘tyrosine cage’ and postulated to play a role in the proper binding of the antigen (Margreitter *et al.*, 2016). The investigation of the 3H6/2F5 complex crystal structure by Bryson *et al.* (2008) assumed that the contact to 2F5 IgG is predominantly formed by the heavy chain complementarity determining region 3 (CDRh3) of Ab2/3H6. Residue 94h is the last residue preceding the CDRh3. Y102h is a member of the tyrosine cage and forms the last residue of CDRh3, suggesting that the tyrosine cage and the interaction between R94h and Y102h may play a role in the proper orientation of CDRh3.

Transient gene expression (TGE) in HEK293-6E suspension cultures can be robustly applied to generate appropriate amounts of recombinant protein, even though the transfection success depends on several factors, such as vector design, transfection reagent and media selection (Meissner *et al.*, 2001). Furthermore, it was shown that the primary amino acid sequence contributes to the performance of the expression host concerning growth rates and specific productivities (Mader *et al.*, 2013). Even minor substitutions or a point mutation in the primary amino acid sequence can significantly affect the expression level (Dueñas *et al.*, 1995; Kipriyanov *et al.*, 1997). Favorable combinations of primary sequences can be related with human germline residues or frequent amino acids at structurally important positions (Hurle *et al.*, 1994; Chromikova *et al.*, 2015). Therefore, the choice of human-to-mouse backmutations is not only relevant for restoring antibody binding, but also to retain or even enhance antibody expression (Popovic *et al.*, 2017). Until now, it is still not possible to fully estimate the effect of point mutations on antibody expression.

In this study, alanine-scanning mutagenesis (Cunningham and Wells, 1989) was used to investigate the role of the tyrosine residues which are involved in the tyrosine cage formation. In addition, arginine at position 94h was substituted by lysine as it is equivalently positively charged. To further enhance the binding affinity of BM07 (T94hR), three new variants with mutations in the variable light chain were defined. Positions 46l and 49l in the light chain were selected for a human-to-mouse backmutation based on their spatial proximity to the CDRh3 loop of the variable heavy chain.

## Materials and Methods

### Transient protein expression of mAb variants

Antibody variants were produced in HEK293-6E host cell line (NRC Biotechnology Research Institute) (Durocher *et al.*, 2002) by co-expression of two different pCEP4 vectors (Invitrogen, #V044-50) with integrated heavy or light chain. Host cells were cultivated in HyClone^TM^ CDM4HEK293 media (GE Healthcare, #SH30858.02) supplemented with 4 mM l-glutamine (Roth, #9183.1), 15 mg/L phenol red (Sigma-Aldrich, #P0290) and 25 μg/mL G418 (Biochrom, Cat. No. A2912) in 125-mL Erlenmeyer flasks (Corning, #431143) in a climo-shaker ISF1-XC (Kuhner) at 150 rpm, 37°C, 7% CO_2_ and 80% humidity.

HEK293-6E cells were transiently transfected with 1 μg of heavy and light chain plasmid per 10^6^ cells with linear 40-kDa polyethylenimine (PEI MAX) (Polysciences, #24765). The transfection mix was fed with 0.5% tryptone N1 (TN1; Sigma Aldrich, #T9410) (Pham *et al.*, 2005) and 5 mM valproic acid (VPA; Sigma Aldrich; dissolved in deionized water, #P4543) (Jäger *et al.*, 2013) 48 h post transfection. Culture supernatants were harvested when viability dropped below 60%.

### Preparation of Ab2/3H6 variants

Culture supernatants were concentrated with Amicon Ultra Centrifugal Filters (0.5 mL, NMWCO 10 kDa, Millipore, #UFC501096). Variants were purified by protein A affinity chromatography using the ÄKTA start system (GE Healthcare) equipped with HiTrap MabSelect SuRe protein A column (GE Healthcare, #29-0491-04).

### Affinity determination of Ab2/3H6 variants

Affinity evaluation of all variants was done by bio-layer interferometry with the FortéBio Octet QK^e^ system (Pall FortéBio) using protein A (Pall FortéBio, #18-5010) or streptavidin biosensors (Pall FortéBio, #18-5019). The baseline steps and sample dilutions were performed in kinetics buffer (FortéBio, #18-5032). In the protein A approach concentrated cell culture supernatants were directly applied and monoclonal antibodies (mAbs) were captured on protein A biosensors, blocked with an unspecific scFv-Fc antibody and the association and dissociation of 2F5 IgG was measured (Margreitter *et al.*, 2016). For the streptavidin approach, the culture supernatants were purified and association/dissociation on streptavidin/biotin immobilized 2F5 was determined to evaluate the affinity. 2F5 IgG was biotinylated with EZ-Link NHS-PEG4-Biotin kit (Thermo Scientific, #21329) and loaded on a streptavidin biosensor.

### Molecular dynamics simulations

Models of the antibody variants were based on the X-ray structure with protein databank (PDB) entry 3BQU (Bryson *et al.*, 2008) as described previously (Margreitter *et al.*, 2016). Molecular dynamics simulations were performed using the GROMOS11 software package, (Schmid *et al.*, 2012) using the GROMOS force field, parameter set 54A8 (Reif *et al.*, 2012). In short, at least four replicate simulations of 50 ns each were performed for all variants. Simulations were performed in explicit solvent (SPC water) (Berendsen *et al.*, 1981) under periodic boundary conditions and at a constant temperature of 300 K and a constant pressure of 1 atm (Berendsen *et al.*, 1984). All bonds were constrained to their optimal bond lengths using the SHAKE algorithm (Ryckaert *et al.*, 1977), allowing for a time step of 2 fs. Coordinates were stored every 2 ps for further analyses. A more detailed description of the simulation protocol can be found in Margreitter *et al.*, 2016.

Hydrogen bond occurrence was monitored using a geometric criterion. An H-bond was observed if the hydrogen-acceptor distance is <0.25 nm, and the donor-hydrogen-acceptor angle is more than 135°. Similarly, parallel stacking interactions between the sidechain of R94h and the individual members of the tyrosine cage are observed if the distance between the centers of geometry of the sidechains’ planar groups are within 0.5 nm and the angle between the planes is at most 30° (Flocco and Mowbray, 1994).

The sampled protein conformations were clustered based on the root-mean-square deviation (RMSD) of all atoms in the CDRh3 (residues 95–102) after a rototranslational fit on the backbone of the flanking framework regions. We focus on the conformations of CDRh3, as this was observed to be the most relevant loop for antigen binding (Bryson *et al.*, 2008; Kunik and Ofran, 2013; Tsuchiya and Mizuguchi, 2016). Conformations with an RMSD below 0.2 nm were considered structural neighbors, and the clustering of conformations is performed as described by Daura *et al.*, 1999, defining a central member structure (CMS) for every distinct cluster of conformations.

## Results

### Selection of heavy and light chain mutants

As a starting point of all herein described mutants we used the mouse-derived superhumanized 3H6 (su3H6) (Mader and Kunert, 2010) which lost antigen binding in the course of humanization. One single back-to-mouse mutation, mutant BM07 in Margreitter *et al.* (2016) (T98hR further referred to as T94hR), restored the binding affinity to the antigen 2F5 partly. Molecular dynamics (MD) simulation suggested an interaction of the charged side chain of R94h with the side chains of Y27h, Y32h and Y102h and the backbone of T95h. T95h and Y102h were not modified as they are part of the CDRh3 loop and therefore considered as crucial for binding (Bryson *et al.*, 2008). Y27h and Y32h were subjected to alanine-scanning mutagenesis to explore the contribution of the so-called tyrosine cage to the conformation of the CDRh3 loop. Therefore, a double mutation variant BM09 (T94hR, Y27hA) and a triple mutation variant BM10 (T94hR, Y27hA, Y32hA) were created. Besides, the role of R94h in BM07 was investigated by an isopolar substitution of residue 94h to lysine (T94hK, BM11). At neutral pH, lysine exhibits a positively charged moiety at a comparable distance to the backbone as arginine. All three heavy chain variants (BM09, BM10, BM11) employ the variable light chain of su3H6.

**Fig. 1:**
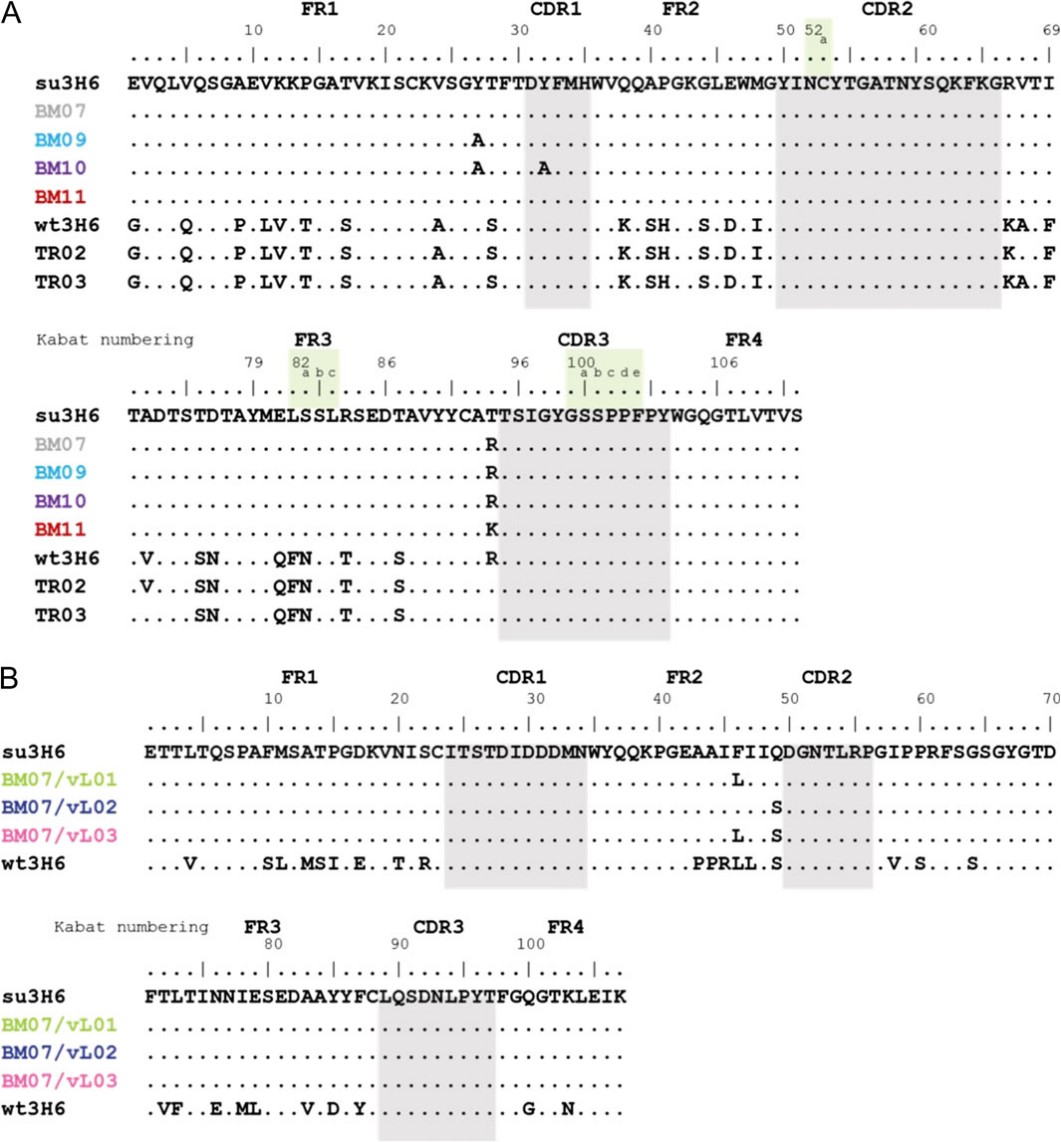
Amino acid sequence of (**A**) variable heavy chain and (**B**) variable light chain of su3H6, su3H6 variants (BM), wt3H6 variants (TR), BM07/vL variants and mouse antibody Ab2/3H6 (wt3H6). Kabat numbering scheme is used and the CDR regions are defined by Kabat using the abYsis tool (Kabat *et al*., 1991; Swindells *et al*., 2017). CDRs are highlighted in gray. Corresponding light chain for variants in panel A is su3H6, respectively, except for TR02/TR03 which have a wt3H6 light chain. BM07/vL variants in panel B use the BM07 heavy chain.

To further enhance binding affinity, three new variants with back-to-mouse mutations in the superhumanized variable light chain were introduced to BM07. These backmutations were selected based on an analysis of the light chain sequences (Fig. 1B) and on their spatial vicinity to the CDRh3 in the available X-ray structure of wt3H6. Double backmutation variants BM07/vL01 (T94hR, F46lL) and BM07/vL02 (T94hR, Q49lS), as well as the triple backmutation variant BM07/vL03 (T94hR, F46lL, Q49lS) were designed. These back-to-mouse mutations involve large or polar amino acids (F, Q), which are exchanged to amino acids with smaller side chains and similar polarity (Bryson *et al.*, 2008). We include two double mutation variants TR02 (wt3H6 + R94hT, A68hV) and TR03 (wt3H6 + R94hT, V72hA) described in Margreitter *et al.* (2016) as references in our simulation experiments since both variants have a threonine in position 94h, as in su3H6. Sequences of all Ab2/3H6 variants are summarized in Fig. 1.

### Molecular dynamics simulations of selected mutants

Molecular dynamics simulations were performed to analyze the selected variants for different parameters: (i) the distances of residue 94h to Y27h, Y32h and Y102h in the tyrosine cage, (ii) the hydrogen-bonding interactions of residue 94h and (iii) the stacking between the planes of the aromatic side-chains of tyrosines and the amino acid at position 94h. All three properties describe possible interactions that may be responsible for conformational changes or maintaining a proper binding structure. The movement of residue 94h was visualized by superimposing snapshots sampled every nanosecond from the simulations. Finally, the CDRh3 loop structure was clustered to identify the main conformations in the various simulations.

#### Position 94h and the tyrosine cage

The distances between the center of geometry of the aromatic ring of each Y residue (Y27h, Y32h and Y102h) and the central carbon, nitrogen and oxygen atom of residue 94h were monitored. Distributions of observed distances are given in Fig. S1 of the Supplementary Material. Distances of less than 0.4 nm were found in mutants with an arginine at position 94h (wt3H6, BM07, BM09, BM10, BM07/vL01, BM07/vL02 and BM07/vL03). In contrast, variants with the smaller amino acid threonine (su3H6, TR02, TR03) or lysine (BM11), show an increased distance to all three tyrosines. We suggest that with even higher distances to all three tyrosines, the tyrosine cage disintegrates as shown in BM11. By investigating BM09 and BM10 for their contribution to the geometry of the modified tyrosine cage we found an overall increased distance of Y102h to R94h. This indicates that the absence of Y32h and/or Y27h reduces the stability. From distance analysis we conclude that the tyrosine cage remains stable in the presence of T94h and R94h, but not with K94h.

We looked at the overall H-bond occurrence of residue R/K/T94h (Table SI). In wt3H6, R94h mainly forms H-bonds to D31h or Y27h. Y27hA mutants like BM09 and BM10 have a higher occurrence of H-bonds between T28h and R94h, which is rarely seen in any other variant. The Y27hA mutation might enable the interaction with residue 28h, which could further result in a restricted motion. H-bonding to Y32h is exclusively seen in BM11, where we have a lysine at position 94h.

Parallel stacking conformations of R94h and the aromatic side-chains of the tyrosine cage is described by the percentage of monitored simulation time in this conformation as illustrated in Fig. S2 in Supplementary Material. Each stacking arrangement, Y27h-R94h, Y32h-R94h and Y102h-R94h, takes place in the vH of wt3H6, BM07, BM07/vL01, BM07/vL02 and BM07/vL03 for 5–35% of the time. Of particular note is that no stacking between Y102h and R94h is observed in BM09 and BM10, although Y102h is not mutated there. Stacking interactions between R94h and Y102h may help to stabilize the appropriate conformation of CDRh3 for antigen binding.

**Fig. 2:**
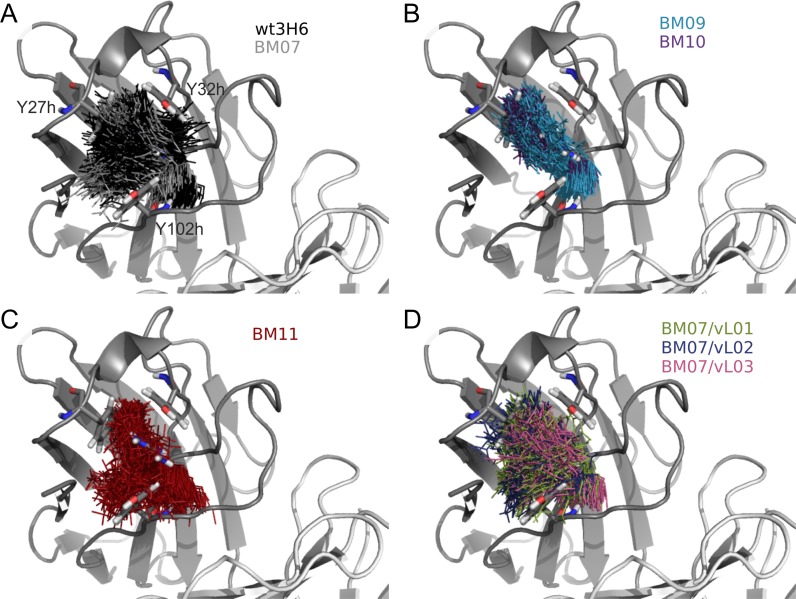
Snapshots (1 per ns) of arginine or lysine at position 94h visualizing the flexibility of residue 94h in the variants. (**A**) black, gray = wt3H6, BM07; (**B**) blue, purple = BM09, BM10; (**C**) red = BM11 and (**D**) green, blue, pink = BM07/vL01, BM07/vL02, BM07/vL03. The cartoon and sticks are from the wt3H6 crystal structure.

Superimposition of one snapshot per ns of R/K94h onto the initial structure of wt3H6 illustrates the clustering of conformations of individual variants (Fig. 2). The sidechain of R94h exhibits a relatively large flexibility in wt3H6 and BM07, which seems to be impaired in BM09 and BM10 by destroying the tyrosine cage. R94h conformations in BM09 and BM10 cluster in a narrow, elliptical cloud which fits with the increased overall distance of R94h to Y102h and/or Y32h (Fig. S1). The reduced flexibility of R94h in BM09 and BM10 is also in agreement with the enhanced H-bonding of R94h to T28h and additionally with the loss of stacking conformation of R94h and Y102h (Fig. S2). BM11, with the lysine at position 94h, and BM07/vL variants show a comparable snapshot cloud as wt3H6 and BM07.

#### Effect of mutations in the variable light chain

Analysis of H-bond formation between residue 49l in the variable light chain showed that H-bonds are mainly observed to neighboring residues 51–53. The back-to-mouse mutation S49l forms significantly more H-bonds to the side-chain T53:Oγ1 than Q49l (Table SI). The H-bond between S49l and T53l stabilizes the turn between two of the variable light chain beta strands and may thereby contribute to the overall stability of the variable domain in the BM07/vL variants. There is no interaction of Q49l with the variable heavy chain observed.

#### The conformation of the CDRh3 loop

**Fig. 3:**
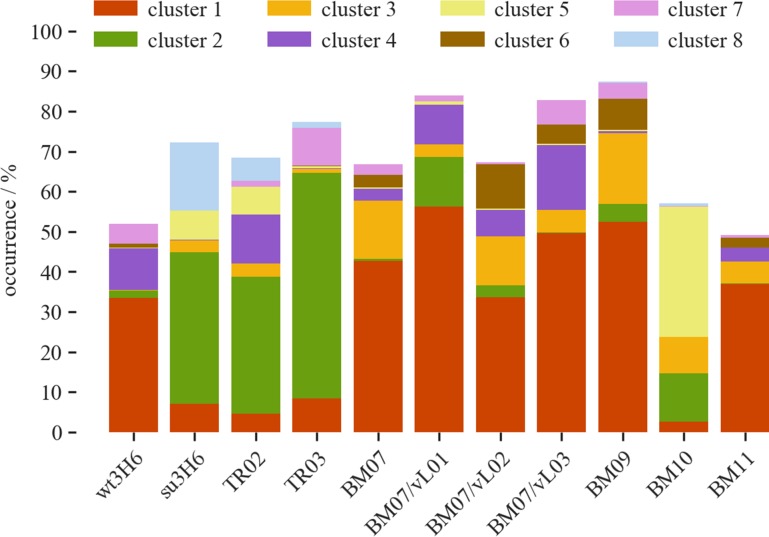
Clusters of CDRh3 loop structure for wt3H6, su3H6, TR02, TR03, BM07, BM09-11 and BM07/vL01-03 including only clusters with overall occurrence >2%.

**Fig. 4:**
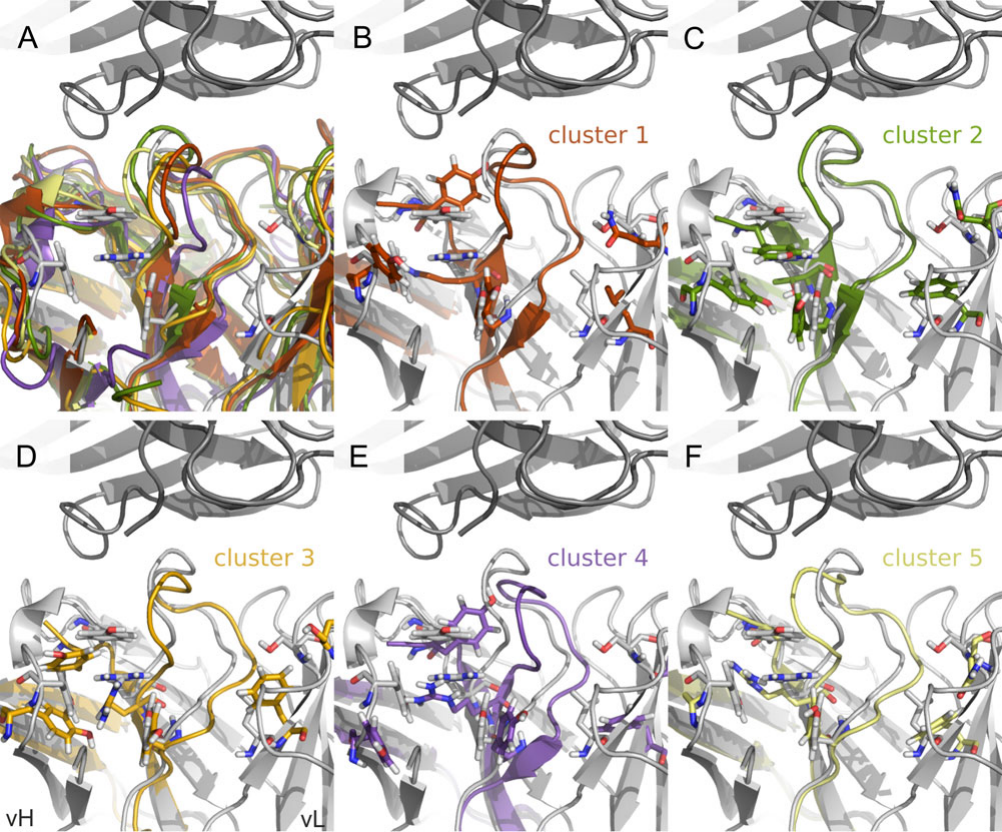
The CMS structures of CDRh3 loop clusters. (**A–F**) In dark gray on top: 2F5, in light gray below: 3H6, both from the crystal structure. The CDRh3 loop is at the center of the picture, left of it is vH, right of it is vL. In (A) the first five CMS structures are overlaid on top of the crystal, in (B–F) CDRh3, FRh3 and FRh4 regions of the five CMS structures are shown individually. The ‘tyrosine cage’, R/K/T94h and F46lL + Q49lS are shown as sticks. Colors correspond to the CDRh3 clusters in Fig. 3.

While it is challenging to predict loop conformations of the CDRs *ab initio* (Kuroda *et al.*, 2012), the current work starts from the X-ray structure of wt3H6 in which the average loop conformation is observed (Bryson *et al.*, 2008). In the simulations different shifts in the conformational ensembles were observed. A joint conformational clustering of all simulations of variants allows us to identify the most important conformations, represented by the CMS of a cluster. Furthermore, we can use the clusters to identify common conformations of the CDRh3 between the variants. We analyzed the clusters with an overall occurrence time of at least 2%, resulting in eight clusters. The occurrence of each cluster is shown in Fig. 3, represented by different colors. The CMS structures of the clustered CDRh3 loop are shown in Fig. 4 in corresponding colors. Figure 3 demonstrates that the most abundant cluster 1 is mainly occurring in wt3H6, BM07, BM09, BM11, BM07/vL01, BM07/vL02 and BM07/vL03. Cluster 2 can only be observed in variants with a threonine at position 94h: su3H6, TR02 and TR03. In this cluster the CDRh3 loop shows a shift towards vH (green, Fig. 4C). Remarkably, BM10 exhibits primarily cluster 5 which is rarely seen in other Ab2/3H6 variants. Absence of the tyrosine cage in BM10 changes the shape of the CDRh3 loop and shifts it towards vH (yellow, Fig. 4F). In contrast to BM07, the BM07/vL variants exhibit a higher abundance of cluster 4 which is the second most relevant cluster for wt3H6. Cluster 1 (red, Fig. 4B) and cluster 4 (purple, Fig. 4E) which are mainly occurring in wt3H6 and BM07/vL variants show a CDRh3 loop close to the X-ray structure (in gray), or slightly moved towards vL.

### IgG-expression

#### Expression of BM and BM07/vL variants

All Ab2/3H6 variants were expressed transiently in HEK293-6E cells. Parameters like total cell concentrations and end-titers differed significantly between the mutated variants which are commonly accepted to be influenced by the expressed antibody (Bentley *et al.*, 1998). To ensure that these differences are not driven by the transfection method or the reagent, we performed multiple individual transfections for each mutant with different DNA preparations and included a positive transfection control as reference. Table I summarizes the end-titer and qP of TGE experiments of all Ab2/3H6 variants. The replacement of large and polar tyrosine residues by alanine resulted in an increase of product titer in BM09 and BM10. Lowest product concentration was reached in BM11, where we assume unfavorable contribution of the isopolar substitution of residue K94h to the protein structure influencing additionally the assembly potential, which results in a hard-to-express IgG. Light chain variants BM07/vL01, BM07/vL02 and BM07/vL03 show a facilitated IgG production, which is probably due to insertion of smaller residues (L, S) instead of large amino acids (F, Q), allowing for a tighter packing of vH and vL or stabilization of the vL itself by hydrogen bonds.

**Table I.** Transient expression of the variants BM09, BM10 and BM11 as well as the light chain variants BM07/vL01, BM07/vL02 and BM07/vL03 are shown. All values are averages of at least three transient transfections with uncertainties computed as the standard deviation. Single backmutation variant BM07 (T94hR) is a single transient transfection

Variant	Mutations	End-titer (μg/mL)		qP (pg/cell/d)	
BM07	T94hR	2.8	–	0.2	–
BM09	T94hR, Y27hA	9.5	±0.3	0.7	±0.1
BM10	T94hR, Y27hA, Y32hA	15.7	±3.3	0.8	±0.0
BM11	T94hK	2.4	±0.6	0.3	±0.1
BM07/vL01	T94hR, F46lL	32.2	±10.9	2.0	±0.7
BM07/vL02	T94hR, Q49lS	9.4	±2.8	1.2	±0.2
BM07/vL03	T94hR, F46lL, Q49lS	49.9	±5.2	2.3	±0.2

### Binding evaluation of Ab2/3H6 variants

**Fig. 5:**
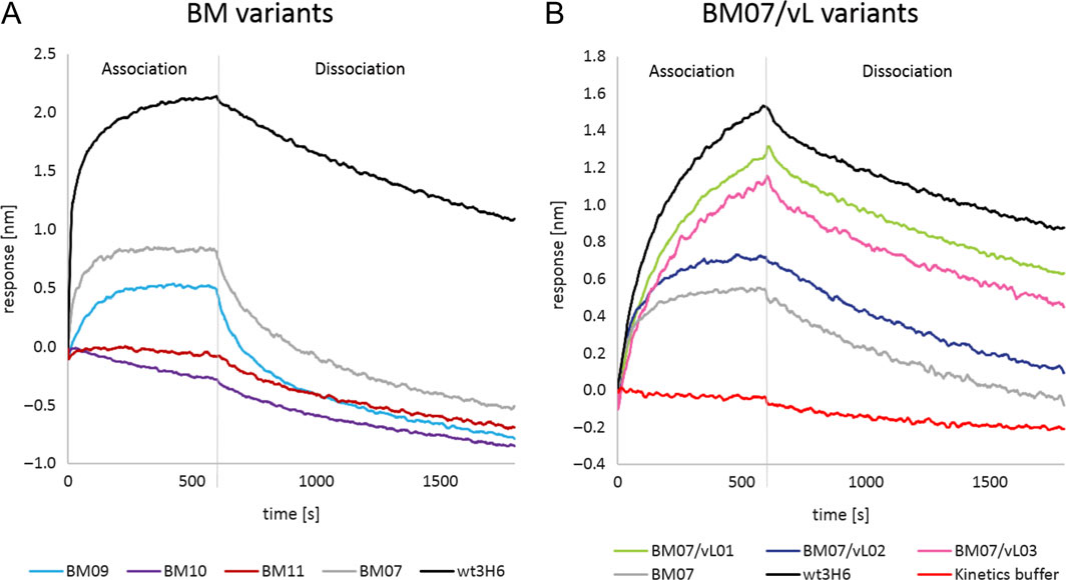
*K*_D_ measurements of Ab2/3H6 variants: real-time bio-layer interferometry (BLI) sensorgram aligned to baseline crude culture supernatant of (**A**) expressed heavy chain BM variants and (**B**) light chain BM07/vL variants detected with 2F5 IgG.

Affinity was evaluated with bio-layer interferometry. Protein A sensor tips as highly sensitive IgG capture step were used to bind Ab2/3H6 variants from concentrated culture supernatants. To analyze the *K*_D_ of the mutants, the potential of association/dissociation of 2F5 IgG (Margreitter *et al.*, 2016) was measured in the next step. Figure 5 shows baseline corrected interaction of 2F5 IgG with heavy (Fig. 5A) and light (Fig. 5B) chain mutants. Table II shows the measured *K*_D_ values using both the protein A sensor method and the streptavidin assay. BM09, the single mutation within the tyrosine cage reduced binding of 2F5 IgG compared to BM07 while the exchange of both tyrosines (Y27h, Y32h) erased 2F5 binding completely. The same was true for BM11 with the T94hK exchange. Regarding the BM07/vL variants, an increased affinity for all three variants was measured compared to BM07. Notably, the BM07/vL variants BM07/vL01 and BM07/vL03 exhibit a comparable *K*_D_ (21–22 nM) to the wildtype (wt3H6), indicating that only a single back-to-mouse mutation in the light chain sequence in proximity of the CDRh3 loop improves BM07 significantly (Fig. 5). These results were confirmed with purified variants analyzed in a different bio-layer interferometry sandwich setup by the streptavidin sensor based bio-assay. Differences in the results can be attributed to the differences in the accuracy of the protein A sensor method and the streptavidin sensor method (Table II). The *K*_D_ of 2F5 IgG for its strong ligand N16N (^656^NEQELLELDKWASLWN^671^) is ≈3 nM and therefore seven times stronger than the binding strength of 2F5 IgG for the anti-idiotypic antibodies BM07/vL01 or BM07/vL03 (Parker *et al.*, 2001; Crespillo *et al.*, 2014).

**Table II.** Comparison of experimentally obtained *K*_D_ values for the binding of 2F5 IgG to Ab2/3H6 variants calculated from protein A and streptavidin bio-assay experiments and averaged from measurements with different Ab2/3H6 concentrations

Variant	Protein A bio-assay	Streptavidin bio-assay
	*K*_D_ average [nM]	*K*_D_ average [nM]
BM07	1.7E+02	3.3E+02
BM09	3.9E+04	8.3E+03
BM10	–*	n.d.
BM11	–*	n.d.
BM07/vL01	2.2E+01	2.8E+01
BM07/vL02	9.5E+01	5.9E+01
BM07/vL03	2.1E+01	2.9E+01
wt3H6	n.d.	3.5E+01
su3H6	–*	–*

*No observable binding; n.d., no data available.

## Discussion

In 2014, the WHO revised the International Nonproprietary Name (INN) definitions for naming antibodies to assess them based upon identity with human V gene germline sequences (World Health Organization (WHO), 2014). Although this definition was changed in June 2017 and the nomenclature describing the species (substem B) is abandoned, the debate about antibody nomenclature continues (Parren *et al.*, 2017; World Health Organization (WHO), 2017). Nonetheless, the human germinality content remains an important issue for the choice of a suitable antibody humanization strategy, to avoid immunogenic side reactions (Bruggemann, 1989). Further information concerning species and germinality content is listed in the associated INN publications. The humanization method which results in the highest germline sequence identity is superhumanization, whereby the murine CDRs of the antibody of interest are grafted onto human germline frameworks (FRs) with the same canonical structure class, chosen based on sequence similarity between murine CDRs and human germline CDRs (Chothia and Lesk, 1987; Tan *et al.*, 2002; Hwang *et al.*, 2005). There is still some debate about the usability of germline FRs for antibody humanization. One of the reasons to favor germline FRs over mature FRs might be that any human being expresses non-mutated germline FRs in IgM antibodies, besides allelic variations thereof. Thus, human germline FRs are tolerated comparably to human self-proteins (Williams *et al.*, 2000). So far, Hwang *et al.*, (2005) demonstrated that superhumanization of D1.3 resulted in a minor reduction of affinity compared to CDR grafting (Jones *et al.*, 1986; Riechmann *et al.*, 1988; Queen *et al.*, 1989) utilizing mature FRs.

The aim of this study was to identify the role of the tyrosine cage and residue 94h as well as to further improve binding of BM07 towards wildtype affinity. Therefore, the strategy of alanine-scanning mutagenesis was applied to investigate the roles of the tyrosine residues Y27h and Y32h involved in the tyrosine cage (Cunningham and Wells, 1989). Y32h is part of CDRh1, but X-ray crystallography has revealed that it is not directly involved in interaction with 2F5 IgG (Bryson *et al.*, 2008). Besides, Bernett *et al.*, 2010 claim that CDRs need not be considered as untouchable and hence Y32h was mutated although it is located in the CDRh1 loop.

Loos *et al.*, 2015 showed that tyrosine sulfation can be critical for the potency of mAbs. Prediction of tyrosine sulfation sites using the online tool ‘The Sulfinator’ indicated a potential modification of Y32h in wt3H6 (Monigatti *et al.*, 2002). MS/MS analysis was done to proof whether wt3H6 exhibits this post-translational modification. However, no tyrosine sulfation was detected in the variable heavy and light chain of wt3H6 (Daniel Maresch, personal communication).

### The influence of the tyrosine cage on the structure, the expression and on binding

To further investigate the correlation of wet lab experiments with molecular dynamics simulations, we carried out additional analyses. High occurrence of CDRh3 cluster 1 corresponds to the high *in silico* binding score prediction for BM07, BM09 and BM11 in Margreitter *et al.*, 2016 (Fig. 3). Alanine-scanning mutations of the tyrosine cage demonstrated a reduction of binding affinity in BM09 (T94hR, Y27hA) and a severe loss of binding in BM10 (T94hR, Y27hA, Y32hA). The MD simulations point out that the tyrosine cage does not give conformational restrictions, but rather conformational freedom. Taken together, these data suggest that the interaction between R94h and Y102h helps to stabilize the relevant binding conformation of CDRh3, since Y102h is the last residue of this loop and together with R94h forms the CDRh3 stem. Moreover, it has already been observed that tyrosine has an enormous energetic contribution to antigen binding, probably due to its versatility in facilitating contacts (Collis *et al.*, 2003; Birtalan *et al.*, 2008; Kunik and Ofran, 2013). The tyrosine cage allows R94h to take an appropriate conformation. Mutation of the cage tyrosines results in R94h getting stuck in alternative interactions and losing the stabilizing effect on CDRh3. Further, in expression experiments we realized that the replacement of large and polar tyrosine residues by alanine increased the expression potential of BM09 and BM10. We conclude that the tyrosine cage plays an important role for supporting a correct CDRh3 loop conformation in the variable heavy chain.

### The influence of lysine on the structure, the expression and on binding

Substitution of R94h by lysine in BM11, which contains a positively charged moiety at a comparable distance to the backbone, was not able to mimic its function appropriately. In our transfection experiments, lowest antibody expression is observed in BM11. We suppose that the unfavorable protein structure influences the assembly and therefore result in a hard-to-express IgG. The more detailed analysis performed here shows that the tyrosine cage largely disintegrates, possibly leading to more diverse CDRh3 conformations and a loss of affinity (Fig. S1 in Supplementary Material).

The discrepancy between the prediction in Margreitter *et al.* (2016), in which BM09 and BM11 were assumed to be reasonable binders, the experimental validation in this work, shows that multiple aspects play a role and that predictions based solely on a single score are not likely to capture the complexity of the affinity between molecules completely. Accurate estimates of protein–protein affinity by computational methods are possible, but computationally highly demanding and for a system of this size hardly feasible (Gumbart *et al.*, 2013; Perthold and Oostenbrink, 2017). For this reason, we restrict ourselves to qualitative interpretations of the binding effect and refrain from explicit predictions of the binding affinity.

### The influence of vL variants on the structure, the expression and on binding

The CMS structures of CDRh3 in Fig. 4 show that the predominant clusters of BM07/vL variants (clusters 1 and 4) are shifted towards vL compared to the crystal structure conformation. We assume that this effect is due to the reduced size of leucine compared to phenylalanine (46l). The back-to-mouse mutations in the vL replace phenylalanine and glutamine by the smaller leucine and serine. Moreover, leucine retains the hydrophobicity, but allows for the right conformation of the CDRh3 loop as it is the case for the backmutation of Q49l to serine. Thereby, they facilitate the arrangement of the CDRh3 loop towards the vL. This conformation seems to provide a favorable binding arrangement. CDRh3 of the non-binding variants su3H6 and BM10 is primarily grouped in clusters 2 and 5 where it is shifted towards vH. Therefore, it can be assumed that a CDRh3 shift towards vH is not favorable for the binding to 2F5 IgG. Moreover, we observed that the replacement of large amino acids with smaller neutral residues near CDRh3 improved the expression. This might be due to substitution of large amino acids by smaller residues, reducing the distance between vH and vL domains to form a more compact and stable Fv molecule (Plückthun *et al.*, 1996). Furthermore, the interactions between S49l and T53l stabilize the beta-turn in the framework region of the vL domain by itself (Hutchinson and Thornton, 1994). More stable proteins are commonly observed to have an improved expression yield (Plückthun *et al.*, 1996). Since each antibody variant is expressed with a characteristic efficiency and minor changes in the framework or CDRs can have a major contribution, (Bentley *et al.*, 1998) a rational design approach can not only be applied to improve antibody binding. It can also be utilized to overcome stability issues or expression challenges in antibody manufacturing (Seeliger, 2013; Mason *et al.*, 2014; Seeliger *et al.*, 2015; Popovic *et al.*, 2017). Popovic *et al.*, 2017 showed the improvement of expression by a single mutation identified by an *in silico* structure-based design approach.

Generally, it is assumed that the vL shows less interaction with the antigen than the vH (MacCallum *et al.*, 1996; Almagro, 2004; Bryson *et al.*, 2008; Kunik and Ofran, 2013). Our measurements resulted in an increase of affinity for all three light chain mutated variants with a *K*_D_ comparable to the value observed for wildtype (Table II). In the MD simulations, we observed that this effect is most likely the result of changes in conformational ensemble of CDRh3, due to the mutations in the vL.

Although Queen *et al.* (1989) introduced a humanized antibody with an affinity that was already close to the original wildtype value back in 1989, restoring a loss of affinity after antibody humanization is often time- and resource-consuming. For that reason, reliable identification of appropriate back-to-mouse mutations is highly needed and the choice of the appropriate framework should be well considered. We have restored binding affinity of a superhumanized mAb to a wildtype comparable level by insertion of two back-to-mouse mutations (T94hR, F46lL). Our results indicate that the residues 46l and 49l in the vL influence the CDRh3 conformation and improve expressability significantly. This demonstrates the underestimated role of the vL for the conformation of the CDRh3 loop and the interaction of Ab2/3H6 and mAb 2F5.

Overall, our work demonstrates that a multidisciplinary approach to antibody humanization can guide the development of variants with wildtype affinity. From a bioinformatics analysis of antibody sequences, we have made the step to a structural interpretation of the effect of suggested mutations and confirmed these experimentally.

## Supplementary Material

Supplementary Data

Supplementary Data

Supplementary Data
