# Supplementary figures and images for: Lessons learned from merging wet lab experiments with molecular simulation to improve mAb humanization
Source: Protein Eng Des Sel. 2018 May 11;31(7-8):257–65. doi: 10.1093/protein/gzy009 (PMC6277173; doi:10.1093/protein/gzy009)

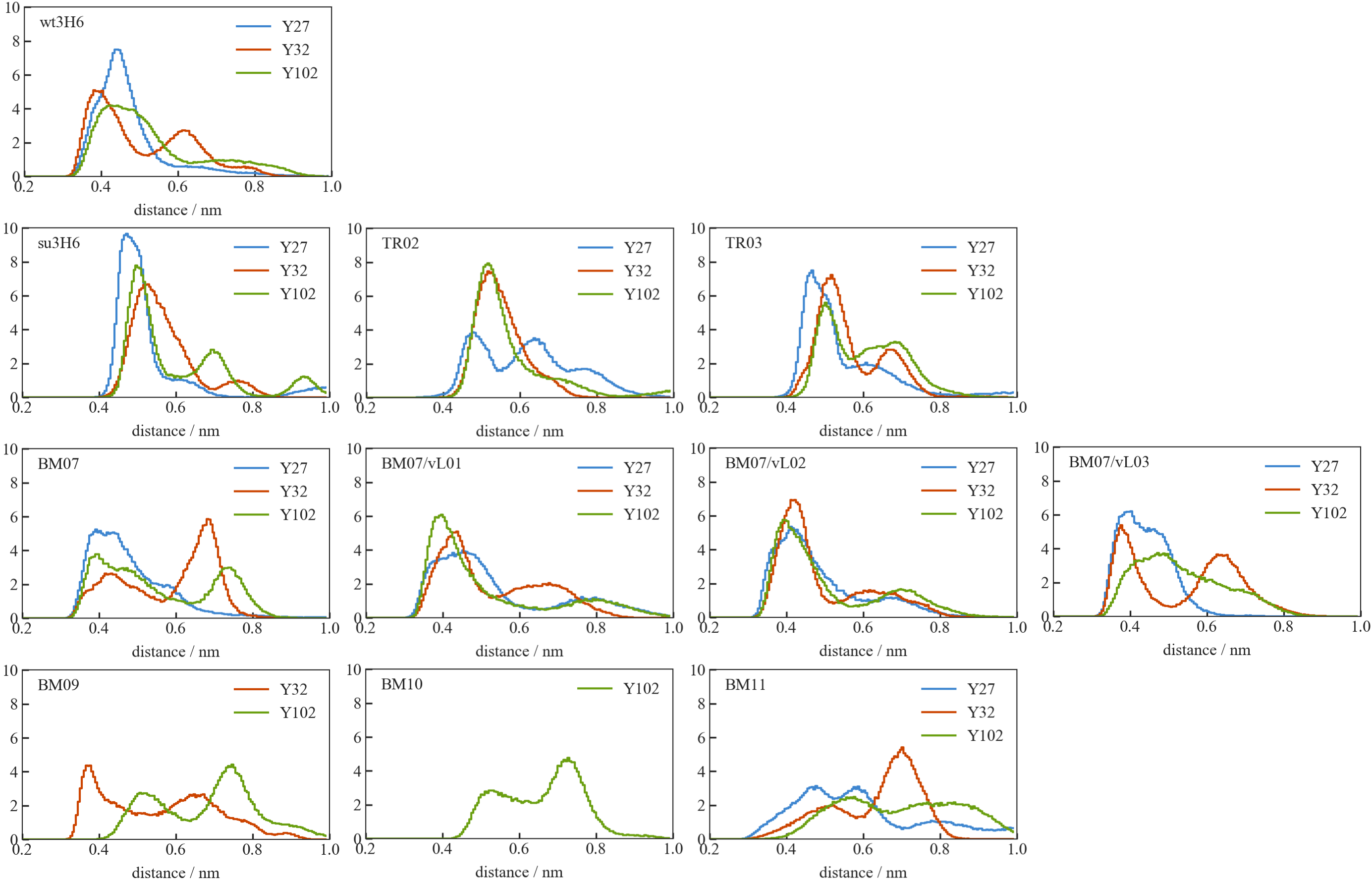

Supplement: Supplementary Data [file figs1.png]

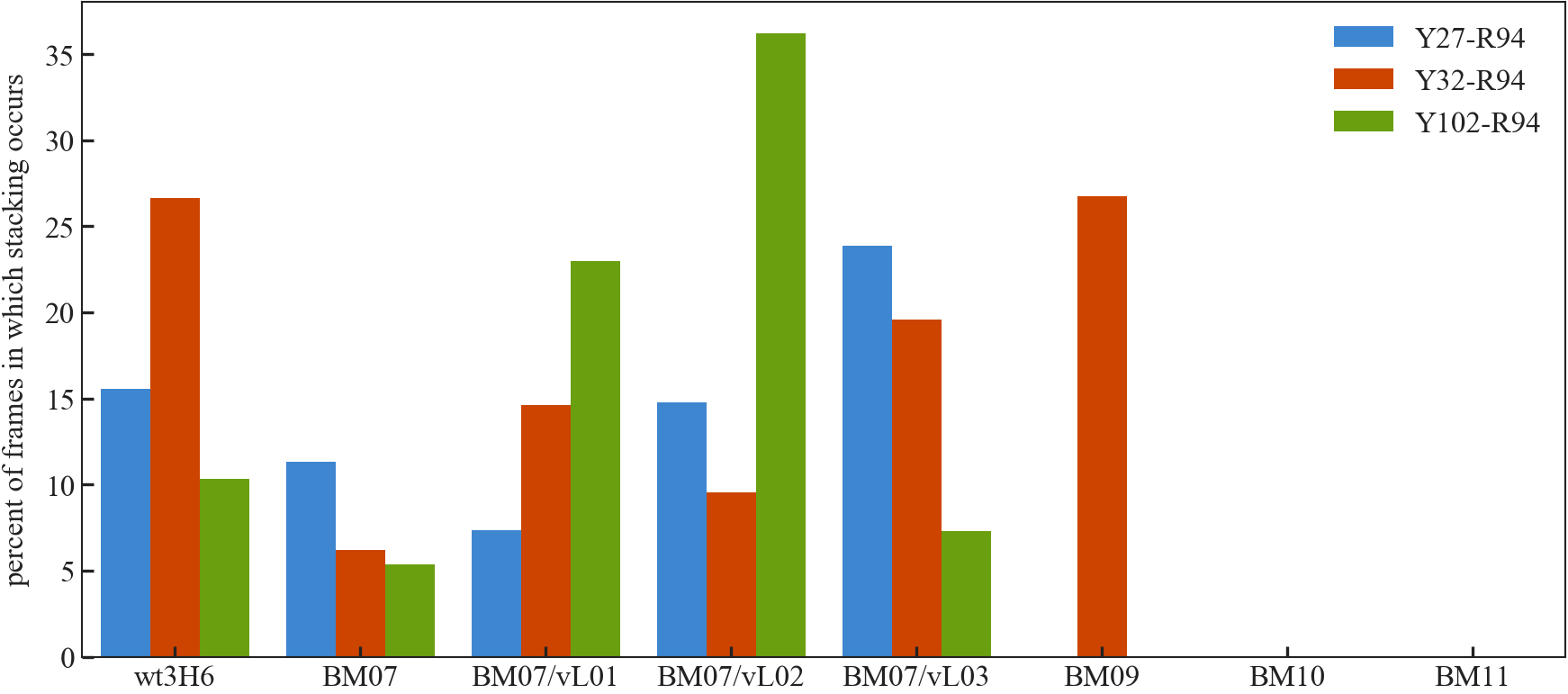

Supplement: Supplementary Data [file figs2.png]
