# Supplementary material for: Lessons learned from merging wet lab experiments with molecular simulation to improve mAb humanization
Source: Protein Eng Des Sel. 2018 May 11;31(7-8):257–65. doi: 10.1093/protein/gzy009 (PMC6277173; doi:10.1093/protein/gzy009)
Supplement: Supplementary Data [file supplementary_material1.pdf]

Supplementary Material

Y-to-R/K/T94h distances

Figure S1: R/K/T94h distances to respective aromatic ring of Y27h, Y32h, Y102h of the ‘tyrosine cage’.

In Figure S1 the distances between the center of geometry of the respective aromatic ring (of Y27h, Y32h, Y102h) and the central atom of residue 94h are shown. If all the distances are short, i.e. all of the tyrosines are close to the central 94h residue, this can indicate a stable tyrosine cage. For variants with arginine (wt3H6, BM07, BM variants, BM07/vL variants) the approximate distance is decreased for all three tyrosines. The most frequent distance of Y27h is mainly occurring at 0.4-0.45 nm and is higher in BM11 with around 0.5 nm. The distance peak of Y32h is in most variants at approx. 0.4 nm, but definitely higher in BM07 and BM11. The distance to Y102h is comparable for all variants, only BM09, BM10 and BM11 show an increased distance, possibly explaining the loss in affinity.

Stacking of R94h and the tyrosine cage

Figure S2: Stacking of R94h and Y27h, Y32h, Y102h of the ‘tyrosine cage’, respectively. Stacking is counted if the distance between the centers of geometry is less than 0.5 nm and the angle between the planes is less than 30°.

H-bond formation

Table S1: H-bonds occurrence between residue 94h and Y/A27h, T/S28h, D31h or Y/A32h in the variable heavy chain. In variable light chain the H-bond formation of Q/S49l with side-chain and backbone of T53h is shown.

Variant	Mutations	Y/A27h – R/K/T94h [%]	T/S28h – R/K94h [%]	D31h – R/K94h [%]	Y/A32h – R/K94h [%]	Q/S49l – T53l:Oγ1 Side-chain [%]	Q/S49l – T53l:O backbone [%]	Q/S49l – T53l:NH backbone [%]
wt3H6	-	21.4	11.1	28.4	3.7	33.5	77.3	31.7
su3H6	-	67.5	0.0	0.0	0.0	12.0	96.2	33.9
TR02	R94hT, A68hV	28.0	0.0	0.0	0.0	-	-	-
TR03	R94hT, V72hA	50.5	0.0	0.0	0.0	-	-	-
BM07	T94hR	8.4	8.7	36.0	1.6	-	-	-
BM09	T94hR, Y27hA	3.7	42.9	13.3	4.5	-	-	-
BM10	T94hR, Y27hA, Y32hA	0.0	33.2	4.7	1.0	-	-	-
BM11	T94hK	20.1	5.4	23.1	27.6	-	-	-
BM07/vL01	T94hR, F46lL	9.6	17.5	5.7	2.0	15.6	94.7	46.6
BM07/vL02	T94hR, Q49lS	40.8	12.2	15.1	4.0	40.7	67.5	64.0
BM07/vL03	T94hR, F46lL, Q49lS	36.7	0.5	28.1	1.0	51.9	88.3	70.9
